# Supplementary material for: Are carbon emissions trading and green financial instruments synergistic? -Comprehensive quantitative research based on content analysis
Source: PLoS One. 2024 Mar 7;19(3):e0298601. doi: 10.1371/journal.pone.0298601 (PMC10919601; doi:10.1371/journal.pone.0298601)
Supplement: S1 Data — (ZIP) [file pone.0298601.s001.zip › Data/地级市Z_F工作报告.zip!/╔╜╬≈/╨├╓▌╩╨/└┤╫╘╣1⁄2╓┌║┼í╛┬φ┐╦╩2╛▌═°í┐_2019.docx]

2018年是全面贯彻党的十九大精神的开局之年，在市委领导下，我们坚持以习近平新时代中国特色社会主义思想为指导，深入贯彻习近平总书记视察山西重要讲话精神，认真落实省委、省政府决策部署，坚持转型升级、脱贫攻坚不动摇，聚焦“三大目标”、“四个重大突破”，深入实施“1661”发展战略，久久为功打基础，孜孜以求谋长远，统筹推进稳增长、促改革、调结构、惠民生、防风险各项工作，保持了经济社会持续健康发展，取得了来之不易的成绩。

　　转型升级初见成效

　　转型项目投资占到固定资产投资的60.2%

　　投资结构发生积极变化。全市转型项目投资占到固定资产投资的60.2%。经济体制实现结构性突破。企业转型升级加快。全市企业技改投资32.5亿元。持续为企业“减、免、缓、帮、扶、替”，规上工业每百元主营业务收入成本降低3.05元，企业利税同比增长43.1%。

　　脱贫振兴统筹推进

　　粮食总产再创历史新高

　　累计整村搬迁763个村，销号458个行政村，安置40464人。城镇化率首次突破50%。繁峙、神池、五寨、岢岚、河曲、保德6个贫困县摘帽进入程序，贫困发生率下降到2.28%。编制了全市乡村振兴战略总体规划和六个专项规划，粮食总产再创历史新高。“中国杂粮之都”建设取得历史性突破。

　　新型综合能源基地建设取得新成效

　　新能源发电装机568.3万千瓦

　　保德煤层气热电联产、海通煤层气液化等一批项目建成或投运，蒙西至晋中特高压交流忻州工程开工。全市电力装机1134.3万千瓦，其中：新能源发电装机568.3万千瓦，火电发电装机566万千瓦，新能源装机首次超过火电规模，实现结构性突破，新型综合能源基地初步形成。

　　产城融合步伐加快

　　城区30条道路竣工通车

　　坚持及时把城市建设成效转化为产业发展成果。中国杂粮产地交易市场开工建设。国家级法兰锻件产品质量监督检验中心正式获批，省级杂粮产品质量检验中心挂牌。忻州城区30条道路竣工通车，云中河景区二期开园，国家卫生城市成果持续巩固，创建全国文明城市通过年度测评，城市综合品质大幅提升。

　　“三个门户”建设对外开放“新高地”作用彰显

　　旅游总收入同比增长23.36%

　　忻州至雄安高铁开展地勘，繁五高速启动招标，繁峙滹源通用机场具备开工条件。神岢高速、晋蒙黄河大桥完工，长城黄河偏关段一号公路开工，同德氧化铝项目重启。大西高铁原太段通车，忻州进入高铁时代。五台山机场航空口岸临时开放，忻州海关获批，机场连接线竣工，永旺保税仓库和出口监管仓库投运，忻州进入口岸开放时代。全域旅游示范区创建取得阶段性成果，旅游总收入同比增长23.36%。

　　重点领域改革不断深化

　　先进产能占比达到75%

　　供给侧结构性改革扎实推进。煤炭退出产能520万吨/年，先进产能占比达到75%。开发区（园区）“三化三制”改革深入推进，繁峙经济技术园区升级为省级开发区，全市开发区新建转型项目数、经营（销售）收入、投资强度、产出强度、税收强度分别完成省定目标的385%、129%、127%、175%、269%。商务综合行政执法体制改革试点评估名列全国第二。

　　生态环保成效明显

　　全市空气质量优良天数比例达到64.4%

　　深入开展大气、水、土壤污染防治“三大战役”。完成“煤改电”、“煤改气”和集中供热等清洁能源替代63691户，全市空气质量优良天数比例达到64.4%，优良天数较2017年增加13天。中央生态环境保护“回头看”交办案件全部办结。关停取缔“散乱污”企业109家，万元地区生产总值能耗下降3.4%。

　　民生事业不断改善

　　市财政民生支出占比80%以上

　　市财政民生支出占比80%以上。2万多名失业人员和就业困难人员实现就业，去产能企业职工妥善安置，城镇新增就业4.61万人，农村劳动力转移就业4.6万人。改（扩）建幼儿园119所。“136”兴医工程全面实施，市儿童医院投运，郭应禄院士工作站在忻州市人民医院挂牌。新建50个农村老年人日间照料中心，省政府确定的六件民生实事全面完成。

　　稳定局面巩固发展

　　连续三年进入中国最安全城市榜单

　　亿元地区生产总值安全生产事故死亡率0.088，控制在省定目标值0.1以内，安全生产事故起数和死亡人数分别下降22.48%、15%。忻州市连续三年进入中国最安全城市榜单，是全省唯一连续三年入榜的市。地方政府债务风险可控，30个项目列入PPP综合信息平台项目库。

　　政府建设不断加强市

　　本级“三公”经费下降7.4%

　　坚持把党的政治建设摆在首位。不断弘扬政府精神，持续建设“六型”政府。全市一般性支出继续压减，市本级“三公”经费下降7.4%。办理9件省人大和省政协建议提案、269件市人大代表建议、335件市政协委员提案，忻州“随手拍”网络问政平台被授予“全国工人先锋号”。深化“放管服效”改革，取消、下放、调整市级行政职权事项153项，清理规范17项行政审批中介服务事项。开展“3545”专项行动，率先在全省将企业开办时间压缩至5个工作日内；扎实推进审批便民服务和“13710”电子督办工作。

　　2019年工作安排

　　2019年是中华人民共和国成立70周年，是全面建成小康社会关键之年。做好今年的政府工作，要以习近平新时代中国特色社会主义思想为指导，全面贯彻党的十九大及十九届二中、三中全会和省委、市委重大工作决策部署，全面实施“1661”发展战略，深入贯彻市委四届六次全会精神，扭住高质量转型发展这个“牛鼻子”，继续打好三大攻坚战；扭住“示范区”、“排头兵”、“新高地”三大目标，统筹推进稳增长、促改革、调结构、惠民生、防风险工作；突出产业升级、转型发展，突出脱贫攻坚、乡村振兴，突出改革创新、开放发展，突出环境保护、生态建设，突出五城联创、改善民生，不断增强人民群众获得感、幸福感、安全感，为全面脱贫全面小康打下决定性基础，以优异成绩迎接中华人民共和国成立70周年。

　　这些成绩的取得，是习近平新时代中国特色社会主义思想科学指引的结果，是省委、省政府和市委正确领导的结果，是市人大、市政协和社会各界监督支持的结果，是全市人民干出来、拼出来的，凝聚了各方面的心血和汗水。当前，我市经济运行稳中有变、变中有忧，突出表现为产业基础薄弱，民营经济发展滞后，创新驱动能力不强，事关项目建设的约束指标基数较低；区域性贫困与深度贫困交织，脱贫攻坚任务艰巨，乡村振兴任务繁重；民生领域存在不少短板，安全生产压力较大，生态环境保护欠账较多；营商环境的基础比较脆弱，吸引好项目、大项目落地的要素不优；政府职能转变不到位，有的政策举措落实还不够精准，部分工作人员工作标准不高。问题是时代的声音。我们要坚持问题起点、目标导向，一切从政府自身找原因，一切通过自身努力推进工作，继续保持一往无前、永不懈怠的工作状态，继续完善求实求是、谋定后动的工作方法，继续营造群策群力、改革创新的工作氛围，继续坚守功成不必在我、功成必定有我的境界和担当，继续保持孜孜以求、久久为功的工作追求，不断增强忧患意识、机遇意识、创新意识，以对忻州高度负责的精神，为我们共同生活的地方发展态势越来越好、创业环境越来越优、人居环境越来越佳而努力，全面拓展在“两转”基础上经济社会和各项事业新局面，扎扎实实办好忻州的每一件事，决不辜负全市干部群众的重托！

　　1扭住发展第一要务不动摇，保持经济持续健康发展

　　全力稳定工业经济运行。加快煤炭减量重组，延长铁矿采选产业链条，提高装备制造科技化水平，增强电力外输能力，实施62个重点企业技改项目，确保工业增加值增长不低于5%、利税增长不低于6%，稳定经济基本面。积极落实国家各项惠企减税降费政策。大力推进科技创新，新增16户企业技术中心，加快6项科技成果产业化，在国家和省级众创空间、科技企业孵化器、高新技术企业建设上再有新突破。

　　千方百计扩大有效投资。加快实施基础设施项目，继续加大交通、物流、市政基础配套设施建设。实施重大基础设施项目90个，年度完成投资117.5亿元。加快大原高铁、冯红铁路建设进度。开工忻雄高铁和朔神、繁五高速，谋划宁静高速，助力太原西北二环静乐段建设；建成长城黄河偏关段一号公路，推进“四好农村路”建设；开工代县航空文化园，加快忻府、原平等通用机场建设前期，推进五台山航空口岸临时开放；建成忻州海关；把5G站址规划、基站、配套纳入市政规划体系。推动重大产业项目落地。着力打造现代煤化、煤电及煤炭物流2个千亿级产业项目集群，全域旅游、煤电铝材一体化、新能源、铁矿采选冶炼、半导体、法兰锻造、煤机装备制造7个百亿级产业项目集群，新型材料、电力装备制造、环保装备制造、生物制药、现代物流、有机绿色杂粮、特色食品加工、特色畜牧、酿酒、通用航空10个十亿级产业项目集群，实施重大产业项目266个，年度计划投资377.8亿元。

　　继续大力招商引资。继续以京津冀、长三角、珠三角为重点，注重以商招商、园区招商、产业链招商，进一步完善招商引资优惠政策，不断提高招商引资的有效性。

　　不断发展民营经济。用足用好用活省市支持民营经济发展两个“30条”意见，认真清理政府部门和国有企业拖欠民营企业账款。着力解决民营企业在立项、环评、要素保障和政策落实中遇到的具体困难。支持民营企业建立研发、营销、管理团队。

　　充分发挥开发区引擎作用。支持忻州经济开发区申报国家级开发区，推动定襄、河曲、岢岚工业园区升级为省级开发区，争取静乐、五台产业园区设立为省级开发区，推动其他县级经济园区申报省级开发区。忻州经济开发区以科技创新为重点、原平经济技术开发区以新型制造为重点、繁峙经济开发区以铸造装备为重点，增强开发区的产业承载力。

　　认真解决具体问题。充分发挥固定资产投资分析调度会、工业经济运行分析会的作用，盯住现有行业、企业存在的问题，加强专项调度，解决生产之忧。

　　2坚持产业转型为本，加快产业结构优化升级

　　不断深化“336”布局。继续打造忻定原面向太原都市圈的中部门户、河曲保德面向陕西内蒙的西部门户、繁峙代县面向京津冀的东部门户，建设忻州古城、五台台怀、宁武东寨三个旅游集散地，发展忻定原综合经济，繁峙代县铸造、加工，河曲保德综合能源，神池五寨岢岚偏关综合生态经济，宁武静乐能源、生态，五台文旅康养六大经济板块。

　　改造提升传统产业。完成同煤北辛窑、晋能芦子沟煤矿建设。提高繁峙、代县球团附加值，推动冶金、型材向精密制造延伸。重启同德氧化铝循环产业园区建设，推进国家电投山西铝业煤电铝镁材一体化、潞安集团煤炭清洁高效利用油化电热气资源一体化项目。

　　培育壮大新兴产业。大力发展新一代半导体及信息技术产业。加快北纬38°公司项目，开工元鸿光电PW/PSS项目、东莞晶驰表镜生产制造项目。大力发展新材料产业。提升忻府区金宇煅烧高岭土项目，投运定襄利国磁性取向硅钢等项目。大力发展现代服务业。投运忻州红星美凯龙、田森汇项目。大力发展生物制药产业。加快发展节能环保产业。

　　着力推进新型综合能源基地建设。投运晋能保德王家岭低热值煤发电项目。投运晋北风电基地145万千瓦核准项目，加快忻府区50万千瓦光伏领跑基地和50万千瓦光伏平价上网基地、19.37万千瓦光伏扶贫村级电站项目建设。推进五寨国耀等生物质能发电项目建设。并网国新能源保德燃气发电项目，推进静乐、宁武南区块和深化中石油煤层气保德区块开发。构建光伏全产业链。

　　加快全域旅游示范区建设。推进涉旅文物保护单位两权分离。实施全域旅游发展总体规划。推进景区标准化建设。加快五台山景区创建全国旅游标准化示范单位，顿村、奇村、凤凰山、大营创建国家级旅游度假区，芦芽山景区、河边民俗馆创建国家5A级景区，老牛湾景区、平型关景区等创建国家4A级景区，定襄七岩山、遗山书院等景区（景点）创建国家3A级景区，实现县县有国家A级景区。

　　大力发展特色现代农业。开展中国特色农产品（杂粮）优势区创建，实施农牧交错带草畜一体化工程，实施加工业提档升级工程，实施质量兴农品牌强农工程。加快实施与山西中医药大学战略合作协议。继续发展有机旱作农业。全年粮食总产稳定在30亿斤以上。

　　深化供给侧结构性改革。引深煤炭“减、优、绿”专项行动，加快化解房地产及非住宅库存，加大“忻材忻用”力度，切实降低企业用电成本。认真落实“巩固、增强、提升、畅通”要求，构建循环畅通的现代产业发展体系。

　　3扎实提高脱贫质量，促进乡村振兴取得新进展

　　主攻贫困摘帽。严格脱贫摘帽标准，补短板、强弱项、创特色，突出抓好脱贫产业培育、村集体经济壮大、基础设施和公共服务改善，实现五台、代县、宁武、静乐、偏关5个县脱贫摘帽。聚焦深度贫困，坚持“一县一策”，细化“10+1”举措，攻克静乐、宁武、偏关3县深度贫困，确保258个贫困村退出、4.5万贫困人口脱贫，实现脱贫攻坚决战决胜。

　　提升脱贫成效。坚持以户为基，着眼六类群体，区别对待，统筹推进，着力在巩固脱贫户、提升边缘户和加快当地产业发展上下功夫，解决收入水平略高于建档立卡贫困户群体的政策支持问题和脱贫户的巩固提升问题，提高帮扶精准度和群众满意度。

　　完善整村搬迁。高质量完成797个村整村搬迁和592个行政村撤并的善后工作，确保新房按时搬入、新区治理完善、旧村及时复垦、每户有人就业“四落实”，推动贫困群众由传统农民向新型居民转变。

　　推动产业发展。继续坚持以户为核心、以合作社为基础、以乡村为依托、以产业为抓手、以政策为杠杆、以发展为根本，抓好家长、社长、村长“三长”和产业体系、政策体系、品牌体系“三个体系”，立足资源禀赋，发挥比较优势，拓展产业扶贫成效。

　　强化兜底保障。完善保障性扶贫举措，健全教育资助政策体系，落实“三保险、三救助”制度，实施好大病救治计划、慢病签约服务、重病兜底保障“三个一批”行动，提升健康扶贫“一站式”结算服务质量，切实打通政策落地“最后一公里”。

　　开展乡村整治。实施贫困村提升工程，加快完善水、电、路、网、房等基础设施，不断优化教育、卫生、文化等公共服务。实施农村人居环境三年集中整治行动，开展“百村示范、千村整治”行动，重点解决好村容村貌问题。

　　优化城乡格局。坚持布局调整为基，统筹推进易地扶贫搬迁与乡村振兴，加快形成以忻定原同城化为核心、县城为区域中心、重要生产经营活动点为基础的新型城乡格局。加快定襄县撤县设区、忻府区3个乡镇改街道办事处进程。

　　4大力促进产城融合，切实加快新型城镇化进程

　　持续改善城市基础设施。以忻州城区北部、东部、南部为重点，新建改造道路32条。北部主攻道路畅通、雨污分流，破解污水处理瓶颈；东部建设东环路，配套电水热气设施，破解产业项目落地的瓶颈；南部配合忻州古城开放，建设东南快速通道，破解由南环路便捷入城的瓶颈。

　　努力夯实宜居宜业基础。紧紧扭住“五城联创”不放，以国家标准倒逼城市功能提升。扩大国家卫生城市创建成果；加快九龙岗森林公园改造，新建城市主题公园，实施顿村植物园扩建工程，完成国家文明城市、国家园林城市创建工作；扎实推进国家智慧城市、环保城市、海绵城市创建工作。

　　不断提升城市管理水平。强化规划管理、秩序管理、土地用途管理、市政管理、园林管理、环卫管理、供热管理、智慧管理、执法管理，不断提升城市建设管理的法治化、精细化、信息化水平。开展道路交通、城乡环境、市场秩序和违法建筑整治，加强建筑施工标准化建设。发挥新组建的城市管理局职能作用，确保城市管理水平有新提升。

　　切实发挥中心城区示范带头作用。瞄准把忻州建成产业新城、文旅名城、田园城市这一目标，驱动城市更新和功能配套，大力提升产城融合水平，切实加快再城镇化步伐。忻州主城区南部建成晋西北生活体验区。忻州古城活化项目7月1日投运。中部建成杂粮产业融合示范区。北部建成温泉康养区。东部建成新兴产业集聚区。用三年时间集中打造成半导体全产业链，推动半导体产业集群发展以及生物制药、现代装备制造业形成规模。
